# Dual RNase and β-lactamase Activity of a Single Enzyme Encoded in Archaea

**DOI:** 10.3390/life10110280

**Published:** 2020-11-14

**Authors:** Seydina M. Diene, Lucile Pinault, Nicholas Armstrong, Said Azza, Vivek Keshri, Saber Khelaifia, Eric Chabrière, Gustavo Caetano-Anolles, Jean-Marc Rolain, Pierre Pontarotti, Didier Raoult

**Affiliations:** 1 MEPHI, IHU-Mediterranee Infection, Aix Marseille University, 19-21 Bd Jean Moulin, 13005 Marseille, France; seydina.diene@univ-amu.fr (S.M.D.); vivek.bioinfo@gmail.com (V.K.); eric.chabriere@univ-amu.fr (E.C.); jean-marc.rolain@univ-amu.fr (J.-M.R.); 2 Assistance Publique-Hôpitaux de Marseille (AP-HM), IHU-Méditerranée Infection, 13005 Marseille, France; lucile.pinault@gmail.com (L.P.); nicholas.armstrong@univ-amu.fr (N.A.); said.azza@univ-amu.fr (S.A.); 3 IHU-Méditerranée Infection, 13005 Marseille, France; saber.khelaifia@univ-amu.fr; 4 Evolutionary Bioinformatics Laboratory, Department of Crop Sciences, University of Illinois at Urbana-Champaign, Urbana, IL 61801, USA; gca@illinois.edu; 5 CNRS, 13005 Marseille, France; pierre.pontarotti@univ-amu.fr

**Keywords:** archaea, metallo-β-lactamases, ribonucleases, glyoxalases, common ancestor sequence, core genes

## Abstract

β-lactam antibiotics have a well-known activity which disturbs the bacterial cell wall biosynthesis and may be cleaved by β-lactamases. However, these drugs are not active on archaea microorganisms, which are naturally resistant because of the lack of β-lactam target in their cell wall. Here, we describe that annotation of genes as β-lactamases in Archaea on the basis of homologous genes is a remnant of identification of the original activities of this group of enzymes, which in fact have multiple functions, including nuclease, ribonuclease, β-lactamase, or glyoxalase, which may specialized over time. We expressed class B β-lactamase enzyme from Methanosarcina barkeri that digest penicillin G. Moreover, while weak glyoxalase activity was detected, a significant ribonuclease activity on bacterial and synthetic RNAs was demonstrated. The β-lactamase activity was inhibited by β-lactamase inhibitor (sulbactam), but its RNAse activity was not. This gene appears to have been transferred to the Flavobacteriaceae group especially the Elizabethkingia genus, in which the expressed gene shows a more specialized activity on thienamycin, but no glyoxalase activity. The expressed class C-like β-lactamase gene, from Methanosarcina sp., also shows hydrolysis activity on nitrocefin and is more closely related to DD-peptidase enzymes. Our findings highlight the need to redefine the nomenclature of β-lactamase enzymes and the specification of multipotent enzymes in different ways in Archaea and bacteria over time.

## 1. Introduction

Antibiotics are part of the microorganism’s arsenal in their struggle to master microbial ecosystems [1]. Most antibiotics are non-ribosomal peptides assembled by megaenzymes, the non-ribosomal peptide synthetases (NRPS) which have structural motifs that appear to be among the oldest living in the world [2,3]. In the case of β-lactams, to date, enzymes named β-lactamases have been identified on the basis of their hydrolyzing activities on this antibiotic family. However, annotation of genomes of multiple living species has shown that homologous sequences to these β-lactamases were present in most living organisms, including those for which there are no known β-lactam targets as seen in bacteria [4]. This is the case in humans where eighteen genes annotated as metallo-β-lactamases have been identified since 1999, some of which, known for their nuclease and/or ribonuclease activities, exhibited indeed a β-lactamase activity [5]. In fact, metallo-β-lactamase (MBL) enzymes are characterized by conserved motif (i.e., HxHxDH) and residues shared by all MBL fold superfamily proteins, including β-lactamases, glyoxalase IIs, nucleases, ribonucleases, and flavoproteins [6,7]. The same is true for Archaea, in which two groups of β-lactamases are present in the majority of Archaea [4], which are not, by nature, susceptible to β-lactams since β-lactams’ target (i.e., the peptidoglycan) is missing in their cell wall, in which an alternative role of these enzymes may be suspected, in particular that of nuclease, ribonuclease, or glyoxalase. Moreover, there is evidence of a transfer event of class B β-lactamase from archaea to a single bacterial group—i.e., the *Flavobacteriaceae* and especially the *Elizabethkingia* genus—which can be responsible for nosocomial infections such as neonatal meningitis, or attack of immunocompromised patients, and has an atypical antibiotic resistance profile, including resistance to thienamycin by an enzymatic mechanism (by expressing GOB and Bla metallo-β-lactamase enzymes) [8,9]. It is highly probable that genes annotated as β-lactamases in Archaea actually support β-lactamase activity in addition to other activities used by Archaea. Thus, in this work, we express identified archaeal class B and C β-lactamases and demonstrate multiple different activities of one archaeal enzyme appearing extremely conserved in archaea.

## 2. Materials and Methods 

### 2.1. Sequence Analysis

A total of 1155 amino acid sequences were retrieved (Class A: 620; B: 174; C: 151, and D: 210) from the ARG-ANNOT database [10]. The phylogenetic trees were inferred using the approximate maximum-likelihood method in FastTree [11]. For a detailed and comprehensive diversity analysis, a few sequences from each clade of the trees were selected as representatives of the corresponding clades (labeled in red in Figures S1 and S2). The ancestral sequence was inferred using the maximum-likelihood method conducted by MEGA6 [12] software. Then, these ancestral sequences were used as queries in a BlastP [13] search (≥30% sequence identity and ≥ 50% query coverage) against the NCBI-nr archaeal database. For Class C β-lactamase analysis, DD-peptidase sequences (penicillin binding proteins) were downloaded from the NCBI database. 2515 sequences were selected for local Blast analysis with the archaeal Class C-like β-lactamase used as the query sequence (gi| 919167542). From this analysis, 24 DD-peptidase sequences were identified as homologous to the query and thus used for further phylogenetic tree analysis. The selected archaeal sequences were aligned with known bacterial β-lactamase sequences (representative sequences of a known clade from the guide tree) using the multiple sequence alignment algorithm MUSCLE [14]. Sequence alignments were then edited with Trimal program to automatically remove spurious sequences or poorly aligned region and the phylogenetic tree was inferred with FastTree using the WAG substitution model and statistical supports of the branch points were tested with the 500 Bootstrap replicates [11]. The inferred tree was then visualized using the interactive Tree Of Life (iTOL v5.) program (https://itol.embl.de).

### 2.2. Antibiotic Susceptibility Testing

*Methanosarcina* (*barkeri* and sp.) isolates were cultured on SAB medium [15] and antibiotic susceptibility testing was performed on this medium for 15 antibiotics including ampicillin, ampicillin/sulbactam, penicillin, piperacillin, piperacillin/tazobactam, cefoxitin, ceftriaxone, ceftazidime imipenem, meropenem, aztreonam, gentamicin, ciprofloxacin, amikacin, and trimethoprim-sulfamethoxazole (I2a, SirScan Discs, France). A filtered aqueous solution of each antibiotic was prepared anaerobically in sterilized Hungate tubes at concentration of 5 mg/mL. Then, 0.1 mL of each of these solutions was added to a freshly inoculated culture tube containing 4.9 mL of the strain tested to obtain a final concentration of 100 µg/mL for each antibiotic tested herein. The mixture of antibiotic and archaeal culture was then incubated at 37 °C and the growth of archaea was observed after 5 to 10 days incubation depending on the tested strain. Control cultures without antibiotic were also incubated under the same conditions to assess strain growth, and non-inoculated culture tubes were used as negative control.

### 2.3. In Vitro Assessment of the β-Lactamase Activity

Protein expression and purification: Genes encoding the selected β-lactamases including the *Methanosarcina* class B β-lactamase protein (gi|851225341), class C-like β-lactamase protein (gi|919167542), and the *Elizabethkingia* GOB-13 (AY647250) were synthesized by GenScript (Piscataway, NJ, USA) and optimized for protein expression in *Escherichia coli* in the pET24a(+) expression vector. The details of this protein expression and purification of the recombinant proteins have been previously described [16]. Briefly, recombinant enzymes were purified using a 5 mL StrepTrap HP column (GE Healthcare) with running buffer 50 mM Tris, 300 mM NaCl at pH 8, and eluted with a step gradient of 2.5 mM desthiobiotin in running buffer. Purified proteins were then subjected to β-lactamase activity detection as previously described using nitrocefin and penicillin in the presence and absence of sulbactam [16]. Furthermore, the activity of MetbaB enzyme was evaluated at different pH (between pH7 and pH10) using the same nitrocefin assay conditions. Kinetic assays were monitored using a Synergy HT microplate reader (BioTek, USA). Reactions were performed at room temperature with a final volume of 100 μL for each well. The nitrocefin substrate was used at concentrations ranging from 0.05 to 1.5 mM, while enzymes were kept at a final concentration of 10 (MetbaB) or 4 µM (Archaeal C-like β-lactamase). Nitrocefin degradation was monitored by measuring absorbance variations at 486 nm. Initial velocities were evaluated by Gen5.1 software and the mean values obtained were fitted using the Michaelis-Menten equation on GraphPad Prism 5 software to calculate kinetic parameters. In addition to performing the analyses described below, the β-lactam hydrolysis activity was monitored by Liquid Chromatography-Mass Spectrometry (LC-MS) on penicillin G and imipenem in the presence and absence of the β-lactamase inhibitor—i.e., sulbactam, as we previously described [16].

### 2.4. Imipenem Antibiotic Degradation Monitored by Liquid Chromatography-Mass

Spectrometry (LC-MS): A stock solutions at 10 mg/ml of Imipenem and Cilastatine was freshly prepared in water from the perfusion mixture of both compounds (500 mg/500 mg; Panpharma, Luitre, France). Amounts of 100 μL of GOB-13 and MetbaB enzyme solutions at 1 mg/ml were spiked with Imipenem/Cilastatine at final concentrations of 10 μg/ml, then incubated at room temperature. Negative controls consisted of PBS spiked with Imipenem/Cilastatine. Triplicate samples were prepared and for each replicate, 30 μL of solution was collected at 0, 4, and 24 h. Then, 70 μL of acetonitrile was added to each sample, and tubes were vortexed for 10 min at 16,000 g to precipitate proteins. The clear supernatant was collected for analysis using an Acquity I-Class UPLC chromatography system connected to a Vion IMS Qtof ion mobility-quadrupole-time of flight mass spectrometer. For each sample stored at 4 °C, 10 μL was injected into a reverse phase column (Acquity BEH C18 1.7 μm 2.1 mm × 50 mm, Waters) maintained at 50 °C. Compounds were eluted at 0.5 mL/min using water and acetonitrile solvents, each containing 0.1% formic acid. The following composition gradient was used: 5% for 1 min to 70% acetonitrile, 95% acetonitrile for a 1 min wash step, and back to the initial composition for 1 minute. Compounds were ionized in the positive mode using a Zspray electrospray ion source with the following parameters: capillary/cone voltages 2.5/40 V, and source/desolvation temperatures 120/450 °C. Ions were then monitored using a High Definition MS(E) data independent acquisition method with the following settings: travelling wave ion mobility survey, 50–1000 m/z, 0.1 s scan time, 6 eV low energy ion transfer, and 20–40 eV high energy for collision-induced dissociation of all ions (low/high energy alternate scans). Mass calibration was adjusted within each run using a lockmass correction (Leucin Enkephalin 556.2766 m/z). The Vion instrument ion mobility cell and time-of-flight tube were calibrated beforehand using a Major Mix solution (Waters) to calculate collision cross section (CCS) values from ion mobility drift times and mass-to-charge ratios. The 4D peaks corresponding to a chromatographic retention time, ion mobility drift time, and parents/fragments masses were then collected from raw data using UNIFI software (version 1.9.3, Waters). As reported, the lactam ring of Imipenem can be hydrolyzed to form the Imipenemoic acid structure. A list of known chemical structures, including Imipenem, Imipenemoic acid, and Cilastatine, was targeted with the following parameters: 0.1 min retention time window, 5% CCS tolerance, 5 ppm m/z tolerance on parent adducts (H^+^ and Na^+^) and 10 mDa m/z tolerance on predicted fragments. Retention times and CCS values were previously measured from antibiotics standards in order to perform subsequent accurate structures screening (Imipenem: 0.3 min/169 Å2; Imipenemoic acid: 1.8 min/242 Å2). The MS Responses of Imipenem and Imipenemoic acid were normalized using the MS Response of Cilastatine (ratio) for data interpretation. Phase I chemical transformations were also screened against the raw data and showed that the hydrolysis (Imipenemoic acid) was an abundant metabolite in the case of GOB-13 and MetbaB.

### 2.5. DNAse and RNAse Activity Evaluation

To evaluate the DNAse activity of the MetbaB enzyme, synthesized single-stranded forward and reverse DNAs and double-stranded DNA of 130-bp were used as substrates. Double-stranded DNA was obtained by annealing forward and reverse single-stranded DNAs in a thermocycler at temperatures decreasing from 95 to 25 °C for 1 h. Moreover, the RNAse activity was assessed using the RNaseAlert QC System kit (Fisher Scientific, Illkirch, France). This assay uses a fluorescence-quenched oligonucleotide probe as substrate that emits a fluorescent signal in the presence of RNase activity. Fluorescence was monitored continuously at 37 °C for 1 h by a Synergy HT plate reader (BioTek Instruments SAS, Colmar, France) with a 485/528 nm filter set. The RNase activity was then determined using supplied RNase A used as a standard (10 mU/mL). In addition to the tested RNaseAlert QC system kit (based on a synthetic RNA), whole *E. coli* RNA was extracted using RNeasy columns (Invitrogen, Carlsbad, CA, USA) according to the manufacturer’s protocol and also tested. Enzymatic reactions were performed by incubating each polynucleotide (DNA or RNA, 1 µg each) with 15 µg of expressed MetbaB protein in Tris-HCl buffer 50 mM, pH 8.0, sodium chloride 0.3 M, using a final volume of 20 µL at 30 °C for 2 h. After incubation, the material was loaded onto denaturing PolyAcrylamide Gel Electrophoresis (dPAGE) at 12% or analyzed using the Agilent RNA 6000 Pico LabChip kit on an Agilent 2100 Bioanalyzer (Agilent Technology, Palo Alto, CA, USA). Of course, RNase activities were assayed in the absence or presence of 10 µg/mL of sulbactam or 10 mM of EDTA or 2.0 U/µL of RNAseOut (Invitrogen, Carlsbad, CA, USA). Negative controls were made with all used reagents (RNase free water, enzyme buffer) but also with bacterial culture without an expression vector containing *metbaB* gene (Blank). Each experiment was performed at least in triplicate.

### 2.6. Glyoxalase II Activity Assay

Glyoxalase II (GloII) activity assays were performed using the Glyoxalase II Activity kit from BioVision (Milpitas, CA, USA) and monitored with a Synergy HT microplate reader (BioTek, Winooski, VT, USA). Reactions were carried out in triplicate at room temperature in a 96-well plate with a final volume of 100 µL for each well. MetbaB was kept at a final concentration of 0.3 mg/mL Degradation of the GloII substrate was monitored for 40 minutes following absorbance variations at 450 nm, corresponding to the production of D-Lactate that reacts with a chromophore provided in the reaction mix. A D-Lactate Standard linear curve was plotted (R2 = 0.9974) and allowed quantification of produced D-Lactate with the MetbaB enzyme and calculation of its specific activity according to the manufacturer’s protocol. The reagent background control (same mixture as in the assay but without any active enzyme) was subtracted from all absorbance measurements.

## 3. Results

Blast analysis of known bacterial β-lactamase genes such as class A (TEM-24, SHV-12), class B (VIM-2, NDM-1), class C (CMY-12, AAC-1), and class D (OXA-23, OXA-58) show no homologous sequences with significant similarities (% identity ≤ 24) against the NCBI archaeal database. However, as described, ancestral sequences are capable of detecting remote homologous sequences from published biological databases [17]. Consequently, using constructed phylogenetic trees of the four bacterial β-lactamase classes, an ancestral sequence for each class was inferred. From the four inferred ancestral sequences, homologous sequences in the archaeal database were identified for the class B and C β-lactamases (Figures S1 and S2 in supplementary). No hits with significant homology were obtained for the class A and D. 

### 3.1. Archaeal Class B Metallo-β-Lactamase 

**Figure 1:**
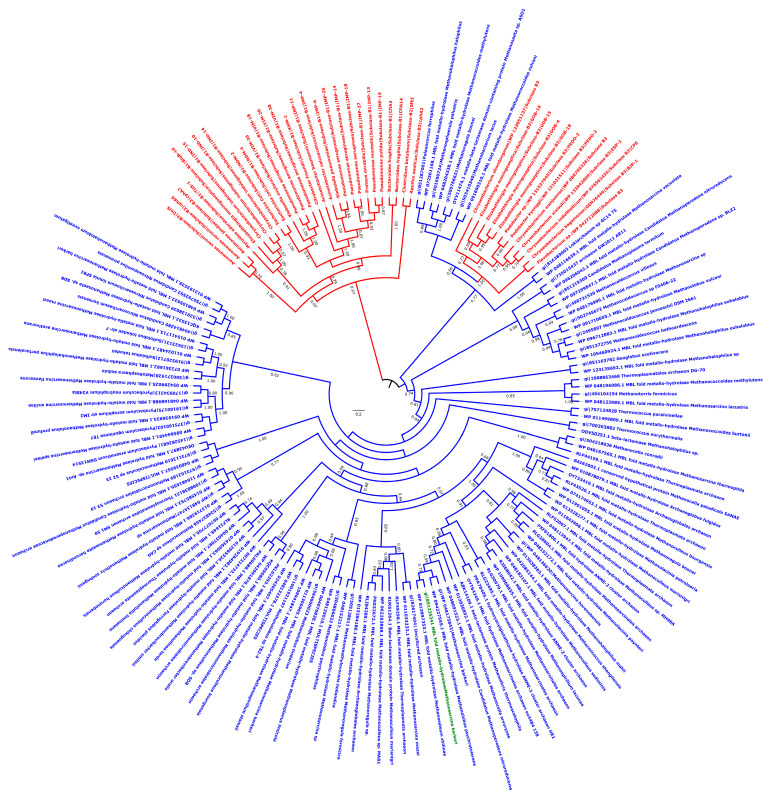
Phylogenetic Tree of Class B β-lactamases from archaea and bacteria. Archaeal sequence colored in green is the one expressed and experimentally tested. Bacterial β-lactamase sequences are colored in red, whereas archaeal sequences are colored in blue.

**Figure 2:**
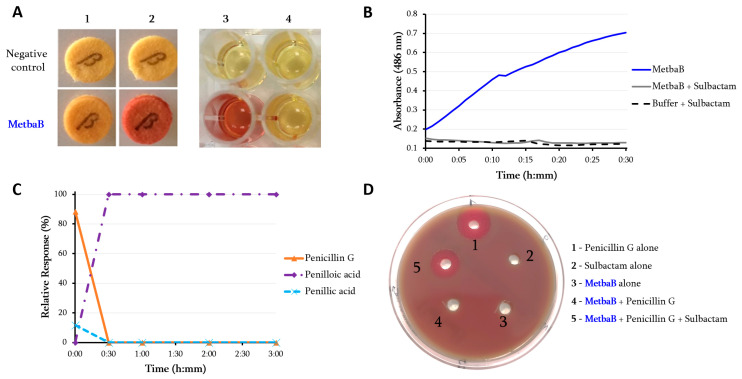
Characterization of the archaeal class B MBL (MetbaB) identified in *Methanosarcina barkeri*. (**A**,**B**): β-lactamase activity of the *M. barkeri* Class B MBL enzyme (MetbaB) on a chromogenic cephalosporin substrate (Nitrocefin). A1 and A2 refer to the nitrocefin degradation test using the BBL™ Cefinase™ paper disc, respectively, at t = 0 and t = 30 min. A3 refers to this same test performed in liquid medium in the absence of sulbactam, while A4, with the addition of 1 µg/ml sulbactam, both after 30 min of incubation; (**B**) monitored nitrocefin degradation by following the absorbance at 486 nm over time in the presence and absence of the β-lactamase inhibitor. (**C**): LC/MS average relative response of screened metabolite compounds of penicillin G in the presence of the *M. barkeri* Class B MBL enzyme, monitored for three hours. Penicillin G (in orange) refers to the intact form of the antibiotic, while penilloic acid (in purple) and penillic acid (in light blue) refer to the penicillin G metabolites. Penicillin G control in PBS did not show any degradation towards any metabolite (data not shown). (**D**) Microbiological test of the mixture of penicillin G (0.1 µg/mL) with the MetbaB enzyme in the presence and absence of sulbactam (15 µg/mL) on a *Pneumococcus* strain highly susceptible to penicillin G (MIC = 0.012 µg/mL) and highly resistant to sulbactam (MIC = 32 µg/mL). The halo around holes 1 and 5 reveals growth inhibition of the *Pneumococcus* strain. The absence of this halo around holes 2, 3, and 4 means that no effect of the mixture on the *Pneumococcus* growth could be observed.

Metallo-β-lactamase fold enzymes appear widely spread in different groups of archaea including *Archaeoglobi*, *Methanomicrobia*, *Methanobacteria*, *Thermococci*, *Methanococci*, *Thermoplasmata, Thermoprotei, Asgardarchaeota*, and *Thaumarchaeota* (Figure 1; Table S1) [4]. To evaluate these archaeal enzymes’ activity, the protein from *Methanosarcina barkeri* (gi|851225341; 213 aa; 25.5 kDa) (Figure 1; Table S1) was experimentally tested. Protein alignment of this latter with known bacterial metallo-β-lactamase proteins reveals conserved motifs/amino acids including Histidine118 (His118), Aspartic acid 120 (Asp120), His196, and His263, markers of this metallo-β-lactamase class B as previously described [18] (Figure S3). Three-dimensional (3D) structure comparison of this enzyme with known and well characterized proteins in the Phyre2 investigator database reveals 100% of confidence and 94% of coverage with the crystal structure of the New Delhi metallo-β-lactamase 1 (NDM-1; Phyre2 ID: c3rkjA) (Table S2). To evaluate these archaeal enzymes’ activity, the MetbaB (*Methanosarcina* β-lactamase class B) protein from *M. barkeri* was experimentally tested. As expected, this enzyme exhibited a significant hydrolysis activity on nitrocefin (Figure 2A,B) (with determined kinetic parameters kcat = 18.2 × 10^−3^ /s, KM = 820 µM and resulting kcat/ KM = 22.19 /ms). Interestingly, in comparison with activities of known bacterial β-lactamases on nitrocefin, these kinetic parameters are comparable with that of CphA β-lactamase enzyme [7]. Moreover, penicillin G hydrolysis was observed by this enzyme when measuring its complete degradation toward a single metabolite—i.e., benzyl penilloic acid—within three hours (Figure 2C). As shown on Figure S4, the MetbaB activity was also evaluated at different pH and was optimal on nitrocefin at pH 7. Furthermore, to confirm the β-lactamase activity of this enzyme, the combination of nitrocefin with β-lactamase inhibitor sulbactam (at 1 µg/mL) was tested. As shown in Figure 2A (column 4), in the presence of sulbactam, no degradation of the nitrocefin β-lactam could be detected, suggesting a complete inhibition of the archaeal β-lactamase enzyme. This neutralizing activity was confirmed microbiologically on a *Pneumococcus* strain highly susceptible to penicillin (MIC = 0.012 µg/mL) and highly resistant to sulbactam (MIC = 32 µg/mL). Indeed, the bacterium could grow in the presence of 0.1 µg/mL of penicillin incubated with the archaeal β-lactamase, but not when sulbactam was added, suggesting an inhibition of penicillin G enzymatic digestion (Figure 2D).

The antibiotic susceptibility testing of a recombinant *E. coli* mutant containing this Archaeal β-lactamase also revealed a reduced susceptibility to penicillin (from 1 to 4 µg/ml) (Figure S5). Interestingly, these Archaeal β-lactamases appear to be closely related to known bacterial metallo-β-lactamase enzymes such as “GOB” type (AF090141), which are fully functional in vivo and found in a single bacterial family—i.e., *Flavobacteriaceae*, especially in the *Elizabethkingia* genus [8,19] (Figure 1 and Figure S6). Indeed, we expressed the *bla*_GOB-13_ gene (AY647250) from *Elizabethkingia meningoseptica* into *E. coli* BL21 strain and detected, by Liquid Chromatography-Mass Spectrometry (LC-MS), a full hydrolysis of imipenem by this enzyme through the accumulation of its metabolite (i.e., imipenemoic acid) over time (Figure S7). As expected, the MetbaB enzyme hydrolyzes also efficiently imipenem since its imipenemoic acid metabolite was detected after 24 h (Figure S7). Specific activities of GOB-13 and MetbaB enzymes were detected in the same order of magnitude on 1 mM of nitrocefin, which were 66 and 24 mU/mg, respectively. 

However, the MBL protein sequences of this bacterial genus compared to those of Archaea revealed low similarities (less than 36%) and this, therefore, suggests an ancient horizontal gene transfer (HGT) from an archaic phylum to this bacterial group, which furthermore exhibited natural β-lactam hydrolysis activity, previously considered to be fairly atypical for a bacterium (Table S3). Therefore, since archaea are resistant to ß-lactams, the role of these β-lactamases in these microorganisms may be the digestion of β-lactams to use them as a carbon source, as reported in the literature on bacteria [20].

### 3.2. Characterization of the DNAse and RNAse Activities 

**Figure 3:**
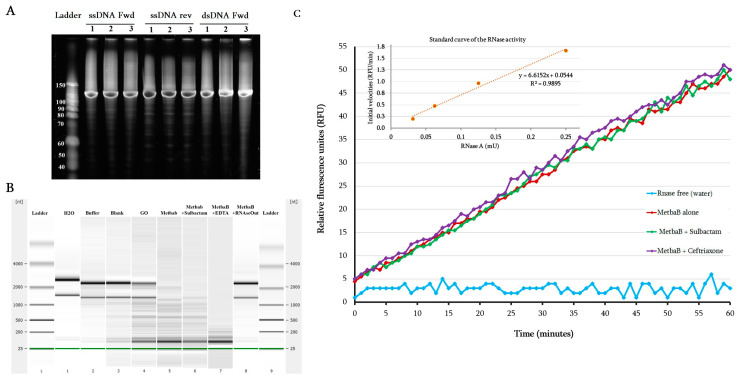
Evaluation of the DNAse and RNAse activity of the archaeal MetbaB enzyme. (**A**) Effect of MetbaB enzyme on different synthetized DNA types (single and double-stranded DNA); each DNA type was tested here three time and no effect was observed. (**B**) Effect of MetbaB enzyme on extracted bacterial RNA (*E. coli* BL21) in the presence and absence of sulbactam (a β-lactamase inhibitor). The bacterial RNA was not degraded when incubated alone with water, enzyme buffer, blank, or with *Bacillus subtilis* Glycine oxidase (GO), an enzyme which is expressed and purified under the same conditions as MetbaB, even if a residual RNA degradation can be observed. In contrast, this bacterial RNA was degraded when incubated with MetbaB enzyme alone or in the presence of sulbactam or EDTA unlike RNAseOut which completely inhibits the RNAse activity. The gels presented in this figure are from different parts of the same gel. (**C**) Confirmation of the RNAse activity of MetbaB enzyme using the RNAseAlert QC system kit. The RNAse activity is estimated according to the accumulated relative fluorescence over time (here, for one hour). The initial volacity of the enzyme was also determied.

As reported in the literature, MBL fold enzymes can have diverse functions such as nuclease, ribonuclease, and/or glyoxalase activities [6,7]. We tested here the nuclease, ribonuclease, and glyoxalase activity of the expressed MetbaB enzyme. As presented in Figure 3A, while no nuclease activity on single and double-stranded DNAs was detected, extracted bacteria RNA (i.e., *E. coli* BL21 strain) was hydrolyzed by the archaeal MetbaB enzyme (Figure 3B). Moreover, using the RNaseAlert QC system kit able to unambiguously detect a real RNAse activity, we were able to confirm the MetbaB RNase activity with an average activity estimated to 0.359 mU/mg ± 0.107 (Figure 3C). Interestingly, inversely to the β-lactamase activity, the MetbaB RNAse activity was not inhibited, neither by the β-lactamase inhibitor (i.e., sulbactam) nor the EDTA chelator, but inhibited by the RNAseOut inhibitor (Figure 3B). As expected, the used negative enzyme control (i.e., Glycine Oxidase (GO)) expressed and purified under the same conditions did not show any RNAse activity.

### 3.3. Glyoxalase Activity 

As presented on Figure S6, the phylogenetic tree analysis shows that glyoxalase II sequences from bacteria and Eukarya appeared to be significantly related to archaeal MBL sequences. Based on this finding, the putative glyoxalase II activity of the MetbaB enzyme was then investigated. We were able to detect a weak activity of 3 mU/mg using the Glyoxalase II activity kit from BioVision (Milpitas, CA, USA) (Figure S8).

### 3.4. Archaeal Class C-Like β-Lactamases 

**Figure 4:**
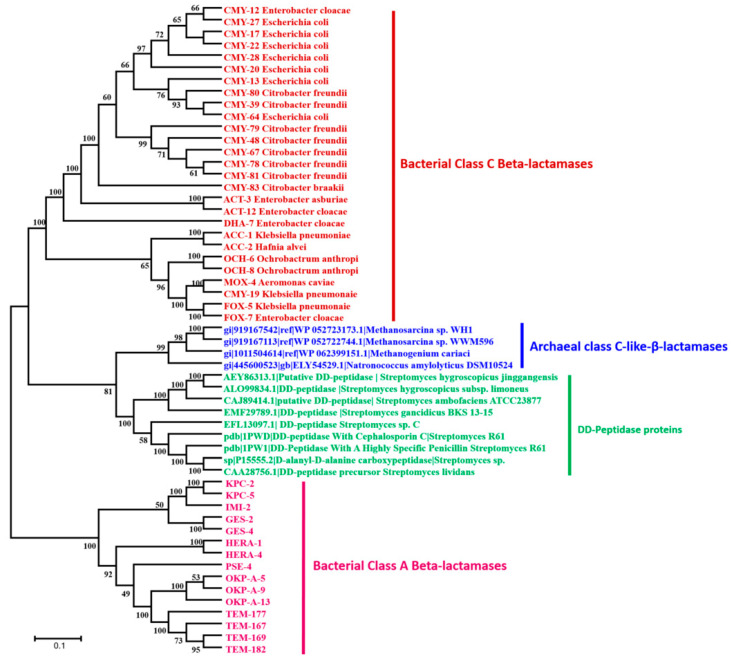
Phylogenetic Tree of Class C β-lactamases and DD-peptidases proteins (penicillin binding proteins). The class A β-lactamases is used as root.

Four homologous sequences with significant similarities to bacterial class C β-lactamase sequences were identified in archaea database using the inferred bacterial class C ancestor sequence (Figure 4; Table S1). The phylogeny analysis shows that this third-class C-like of β-lactamases appears to be a very old class, a putative new clade, which cannot be identified without the reconstruction of the common ancestor (Figure 4). As shown in this figure, this class C-like enzyme appears to be more closely related to DD-peptidase enzymes than the known bacterial class C β-lactamases. Protein alignment reveals the same conserved and signature motifs (S64xxK and Y150xN) identified in bacterial class C β-lactamase (Figure S9). Moreover, DD-peptidase enzymes can exhibit significant β-lactamase activity (10-fold higher β-lactams resistance) through point mutations in the coding sequence [21]. The three-dimensional (3D) structure comparison of this archaeal class C-like enzyme with known and well characterized proteins in the Phyre2 investigator database reveals 100% confidence and 66% coverage with the crystal structure of the octameric penicillin-binding protein (PBP) homologue from *Pyrococcus abyssi* (Phyre2 ID: c2qmiH) (Table S2). Similarly, the identified archaeal enzyme of this class C (gi|919167542) was also cloned in *E. coli* and was found to be active at the enzymatic level by hydrolyzing the nitrocefin (Figure S10). This enzymatic activity was also confirmed by the kinetic assays, showing the catalytic parameters kcat = 9.67 × 10^−3^ /s, Km = 583.6 µM, and kcat/ Km = 16.57 /ms, according to Michaelis–Menten equation fitting (R2 = 0.984). However, the β-lactams susceptibility testing of the recombinant *E. coli* strains harboring this sequence reveals no reduced susceptibility, as compared to the control *E. coli* strains.

## 4. Discussion

In this study, our findings suggest that the Archaeal MetbaB enzyme has triple activities, comprising β-lactamase, ribonuclease, and glyoxalase, and the annotation corresponding to only one of these activities is biologically unsatisfactory. The archaea microorganisms, in which the tested β-lactamase was identified (*M. barkeri*), are fully resistant to all β-lactam antibiotics. This archaeal species has the largest genomes in the Archaea kingdom because of a massive HGT from bacteria [22]. The identified class B β-lactamase sequences appear highly conserved and widespread in Archaea as previously reported using the hidden Markov model (HHM)-based profile [4] and sequence transfer events were observed into a single bacterial family, particularly in the *Elizabethkingia* genus, which has one of the largest spectra of resistance to β-lactams known to date. In the current literature emerges evidence that β-lactamase enzymes, especially the class B metallo-β-lactamase superfamily, have various activities such as β-lactamase, nuclease, ribonuclease, and glyoxalase [6,23,24] (Figure S6), which could justify the existence of enzymes in archaea acting also as β-lactamases. It has been reported that MBL enzyme superfamily exhibit a landscape of crossed activities, since each enzyme has on average 1.5 catalytic reactions in addition to its native activity [25]. Here, we were able to demonstrate that expressed archaeal β-lactamase enzyme (class B β-lactamase) can have triple activities (β-lactamase, ribonuclease, and glyoxalase). While the RNase activity was not inhibited, neither by β-lactamase inhibitor (sulbactam) nor EDTA chelator, the antibiotic hydrolyzing activity was inhibited by this inhibitor, a drug commonly used, in the treatment of human infections, to inhibit bacterial β-lactamases [26]. The consequences of inhibiting the activities of these enzymes in the physiology of host organisms is an area that remains to be explored. According to our results, the role of β-lactamase-like enzymes in Archaea does not seem to be fully understood yet. There is confusion between the annotation of nucleases/ribonucleases and β-lactamase enzymes. Both activities can be conserved in archaea and this is likely to demonstrate the ancient origin of MBL nucleases and secondly, the risk of false annotation from the first identified enzymatic activity of the newly identified enzymes. Our findings suggest that archaeal β-lactamases are as ancestral as those of bacteria, and HGT events have occurred from archaea to bacteria, where enzymes can be specialized to other roles for more efficiency, as observed in *Elizabethkingia*. As presented in Figure S11, we propose a putative evolution scenario of enzymatic activities of MBLs in which the ancestor MBL sequence has best hit the protein in Protein Database Bank (PDB), a glyoxalase II (1XM8_A) exhibiting two different metal ions (Fe and Zn) in its catalytic site. Its evolution over time resulted in (i) archaeal MBLs—e.g., MetbaB—which matches in PDB with a MBL superfamily fold (AZZI_A) exhibiting only Fe ion in its catalytic site and has different enzymatic activities, as observed in our present study, and (ii) bacterial GOB enzymes (K0W_A) carrying only Zn ions for a more specific and effective activity against β-lactam antibiotics. Thus, in future, it would be interesting to investigate the effect of catalytic site inactivation on the different observed activities of this MBL fold enzyme.

## 5. Conclusions

Finally, the existence of enzymes in the world of archaea with multiple activities such as β-lactamase, ribonuclease, and glyoxalase II shows that β-lactamase enzymes are not only a defense system against β-lactam antibiotics. These multiple activities appear as promiscuous activities of these MBL fold enzymes, as suggested in the literature through sequence similarity network analysis [25,27]. Moreover, very recently, RNA-hydrolysis activity of bacterial class B metallo-β-lactamase IMP-1 has been reported experimentally [28]. The use of antibiotics as a source of nutrients for archaebacteria to degrade the molecules of β-lactam and use them as a source of carbon, as described in the bacteria, is a plausible hypothesis [20,29,30,31].

## Supplementary Materials

The following figures are available online at https://www.mdpi.com/2075-1729/10/11/280/s1 Figure S1: Phylogenetic Tree of Class B β-lactamases: the phylogenetic tree was produced using the approximate Maximum Likelihood method under the JTT matrix-based model. The analysis involved 174 amino acid sequences from class B β-lactamases. Evolutionary analysis was conducted in FastTree and visualized in FigTree. The existing clades are labeled as b-1 to b-4. Figure S2: Phylogenetic tree of Class C-like β-lactamases: the phylogenetic tree was inferred using the approximate Maximum Likelihood method under the JTT matrix-based model. The analysis involved 151 amino acid sequences from the class C-like β-lactamases. Evolutionary analysis was conducted in FastTree and visualized in FigTree. The existing clades are labeled as c-1 and c-2. Figure S3: Protein sequences alignment of bacterial and archaeal class B β-lactamases. Known and conserved residues of bacterial metallo-β-lactamases are highlighted with yellow color. Figure S4: Evaluation of the archaeal MetbaB enzyme activity on nitrocefin at different pH. (A) The monitoring of the nitrocefin degradation by MetbaB enzyme for 30 min under the four different pH conditions. (B) The MetbaB activity test on the chromogenic cephalosporin substrate in liquid medium. Figure S5: The antibiotic susceptibility testing of a recombinant *E. coli* BL21 mutant containing this Archaeal β-lactamase (*metbaB* gene). GC: Positive growth control. Figure S6: Phylogenetic relation cheap of different MBL fold proteins including β-lactamases, nucleases, ribonucleases, glyoxalase, and identified enzymes from humans, bacteria, archaea, and giant viruses. Figure S7: Monitoring the imipenem hydrolysis by the *E. meningoseptica* metallo-β-lactamase GOB-13 and MetbaB enzyme by LC-MS. Time depended production of the metabolite of imipenem (i.e., imipenemoic acid) is measured here using an unhydrolyzed cilastatine substrate. Both enzymes hydrolyze efficiently imipenem through the increase accumulation of its metabolite. Figure S8: Glyoxalase II activity assay. D-lactate production was monitored for 40 min following absorbance variations at 450 nm. The GloII positive control was provided in the “Glyoxalase II Activity kit” from BioVision. The negative control was the reagent background control (same mixture with no active enzyme). Figure S9: Protein alignment sequences of bacterial and archaeal class C-like β-lactamase proteins. Known and conserved motifs of bacterial class C β-lactamases are highlighted with yellow color. Figure S10: Degradation of nitrocefin by MetbaB and the archaeal class C-like β-lactamases was monitored for 10 min following absorbance variations at 486 nm. Enzymes were, respectively, kept at concentrations of 10 and 4 µM for this assay. The negative control displayed here was the same reagent mixture without any active enzyme. Figure S11: Putative evolution scenario of enzymatic activities of MBL fold proteins. (+): positive activity; (-): negative activity. Table S1: Best blast hits of ancestor β-lactamase sequence against the NCBI Archaea database. Table S2: Three-dimensional (3D) structure comparison with available and characterized proteins from the Phyre2 investigator database. Table S3: Antibiotic resistance pattern of *Methanosarcina* compared to *Elizabethkingia* GOB-15. Nd—not determined. R, resistance; S, Susceptible. 13 - Opota et al., IJAA 49, 93–97 (2016).
