# Supplementary material for: Dual RNase and β-lactamase Activity of a Single Enzyme Encoded in Archaea
Source: Life (Basel). 2020 Nov 14;10(11):280. doi: 10.3390/life10110280 (PMC7697635; doi:10.3390/life10110280)
Supplement: Supplementary file 1 [file life-10-00280-s001.pdf]

Supplementary

Dual RNase and β-lactamase Activity of a Single Enzyme Encoded in Archaea

Seydina M. Diene [1], Lucile Pinault [2], Nicholas Armstrong [2], Said Azza [2], Vivek Keshri [1], Saber Khelaifia [3], Eric Chabrière [1], Gustavo Caetano-Anolles [4], Jean-Marc Rolain [1,2], Pierre Pontarotti [1,5] and Didier Raoult [1,2,3,*]

[1] MEPHI, IHU-Mediterranee Infection, Aix Marseille Univ, 19-21 Bd Jean Moulin, 13005 Marseille, France; seydina.diene@univ-amu.fr (S.M.D.); vivek.bioinfo@gmail.com (V.K.); eric.chabriere@univ-amu.fr (E.C.); jean-marc.rolain@univ-amu.fr (J.-M.R.); pierre.pontarotti@univ-amu.fr (P.P.)

[2] Assistance Publique-Hôpitaux de Marseille (AP-HM), IHU-Méditerranée Infection, 13005 Marseille, France; lucile.pinault@gmail.com (L.P.); nicholas.armstrong@univ-amu.fr (N.A.); said.azza@univ-amu.fr (S.A.).

[3] IHU-Méditerranée Infection, 13005 Marseille, France; saber.khelaifia@univ-amu.fr

[4] Evolutionary Bioinformatics Laboratory, Department of Crop Sciences, University of Illinois at Urbana-Champaign, Urbana, IL 61801, USA; gca@illinois.edu

[5] CNRS, 13005 Marseille, France; pierre.pontarotti@univ-amu.fr

[*] Correspondence: didier.raoult@gmail.com; Tel.: +33- 4-1373-2401.

Table 1. Best blast Hits of ancestor β-lactamase sequence against the NCBI Archaea database.

Query id	NCBI Hit Gi	Organism Hits	Size (bp)	Descriptions	% Identity	E-value	% Q_Cov
**Ancestor class B β-lactamase**	gi|564600023	*Methanolobus tindarius*	639	Zn-dependent hydrolase	34.62	5.26×10^{-9}	51.17
	gi|503410340	*Methanobacterium lacus*	645	Hydrolase	34.23	0.018	57.75
	gi|504218036	*Methanocella conradii*	624	Hydrolase glyoxylase	33.58	1.17×10^{-6}	53.99
	gi|145282681	*Pyrobaculum arsenaticum* DSM 13514	621	Beta-lactamase domain protein	33.33	2.14×10^{-7}	52.58
	gi|375160102	*Pyrobaculum oguniense* TE7	621	Zn-dependent hydrolase glyoxylase	32.52	8.80×10^{-5}	52.58
	gi|2495897	*Methanocaldococcus jannaschii* DSM2661	618	Probable metallo-hydrolase MJ0296	32.17	1.76×10^{-4}	50.70
	gi|1008838121	*Thermoplasmatales archaeon* SM1-50	651	Hypothetical protein	32.14	0.28	54.46
	gi|82617404	uncultured archaeon	645	Hypothetical protein	32.12	1.82×10^{-5}	53.05
	gi|1008853468	*Thermoplasmatales archaeon* DG-70	606	Hypothetical protein	31.78	0.011	51.17
	gi|851372756	*Methanocaldococcus bathoardescens*	567	MBL fold metallo-hydrolase	31.58	1.05×10^{-4}	50.70
	**gi|851225341**	***Methanosarcina barkeri***	**641**	**MBL fold hydrolase (MetbaB)**	**31.54**	$\mathbf{4.88 \times 10^{-5}}$	**51.17**
	gi|851312700	*Methanosarcina barkeri*	641	MBL fold hydrolase	31.54	2.85×10^{-5}	51.17
	gi|490731539	*Methanocaldococcus villosus*	540	Hypothetical protein	31.53	1.87×10^{-5}	50.70
	gi|1001921197	*Methanolobus* sp. T82-4	672	Hypothetical protein AWU59_1880	31.39	2.10×10^{-6}	53.99
	gi|505138611	*Methanomethylovorans hollandica*	645	Zn-dependent hydrolase glyoxylase	31.39	6.56×10^{-8}	54.46
	gi|501690724	*Methanosphaerula palustris*	696	MBL fold hydrolase	31.37	0.002	56.34

	gi|18160175	*Pyrobaculum aerophilum* str. IM2	624	Possibly metallo-beta-lactamase superfamily	30.89	2.21×10^{-6}	52.58
	gi|494814289	*Candidatus Nitrosoarchaeum koreensis*	611	Zn-dependent hydrolase	30.83	0.029	54.46
	gi|504866623	*Methanolobus psychrophilus*	641	MBL fold hydrolase	30.77	1.73×10^{-5}	51.17
	gi|816389003	*Lokiarchaeum* sp. GC14_75	648	Metallo-beta-lactamase L1	30.77	3.64×10^{-8}	59.62
	gi|851262085	*Methanosarcina horonobensis*	641	MBL fold hydrolase	30.77	1.56×10^{-4}	51.17
	gi|502745672	*Methanocaldococcus* sp. FS406-22	558	MBL fold metallo-hydrolase	30.70	5.98×10^{-5}	50.70
	gi|170934313	*Pyrobaculum neutrophilum* V24Sta	615	Beta-lactamase domain protein	30.65	1.60×10^{-6}	53.05
	gi|757124828	*Thermococcus paralvinellae*	717	Zn-dependent hydrolase	30.61	0.002	55.87
	gi|756792592	*Candidatus Nitrosopumilus piranensis*	1404	Rhodanese domain-containing protein	30.56	2.45×10^{-5}	63.38
	gi|851287001	*Palaeococcus ferrophilus*	609	Glyoxalase	30.51	8.05×10^{-4}	50.70
	gi|973113610	*Methanocalculus* sp. 52_23	603	Beta-lactamase domain protein	30.40	8.24×10^{-6}	51.17
	gi|973162189	*Methanomicrobiales archaeon* 53_19	603	Beta-lactamase domain protein	30.40	6.11×10^{-6}	51.17
	gi|524837456	*Methanoculleus* sp. CAG:1088	641	Putative uncharacterized protein	30.37	0.035	50.70
	gi|851257745	*Methanosarcina*	600	Hypothetical protein	30.37	0.13	59.62
	gi|700303882	*Thermococcus eurythermalis*	681	Hydrolase	30.28	2.08×10^{-4}	52.11
	gi|735015437	archaeon GW2011_AR11	714	Beta-lactamase protein	30.25	0.23	50.70
	gi|15623131	*Sulfolobus tokodaii* str. 7	600	Putative hydrolase	30.23	7.15×10^{-6}	54.46
	gi|500271928	*Metallosphaera sedula*	606	MBL fold metallo-hydrolase	30.23	6.05×10^{-4}	53.52
	gi|919520712	*Sulfolobus tokodaii*	597	Hypothetical protein	30.23	6.99×10^{-6}	54.46
	gi|851163782	*Geoglobus acetivorans*	615	Zn-dependent hydrolase	30.17	1.29×10^{-4}	50.70
	gi|851219309	*Candidatus Methanoplasma termitum*	576	MBL fold metallo-hydrolase	30.08	0.032	52.58
	gi|494104154	*Methanotorris formicicus*	618	Hypothetical protein	30.08	4.44×10^{-6}	53.05
	gi|329138039	*Candidatus Nitrosoarchaeum limnia*	1398	Rhodanese domain-containing protein	30.07	6.43×10^{-7}	63.38
	gi|500766631	*Methanoregula boonei*	696	MBL fold metallo-hydrolase	30.07	0.055	56.34
**Ancestor**	gi|445600523	*Natronococcus amylolyticus* DSM10524	1974	β-lactamase	31.07	1.66×10^{-13}	52.02
**class C**	gi|1011504614	*Methanogenium cariaci*	834	Hypothetical protein	30.80	7.49×10^{-12}	57.68
**β-lactamase**	**gi|919167542**	***Methanosarcina sp.* WH1**	**1959**	**Hypothetical protein**	**30.63**	$\mathbf{1.45 \times 10^{-17}}$	**75.74**
	gi|919167113	*Methanosarcina sp.* WWM596	1959	Hypothetical protein	30.63	1.82×10^{-17}	75.74

Table 2. Three-Dimensional (3D) Structure Comparison with Available and Characterized Proteins from the Phyre^{2} Investigator Database.

Archaea		Phyre^{2} Investigator Database					
**Protein Sequences**	**Size (aa)**	**Best Protein Hit**	**Hit ID**	**Confidence**	**Coverage**	**Comments**	**3D Structure**
**Class B β-lactamase**	**213**	Crystal structure of New Delhi Metallo-β-lactamase (NDM-1) form *Klebsiella pneumoniae*	c3rkjA	100%	94%	201 residues (94%) have been modelled with 100% confidence by the single highest scoring template	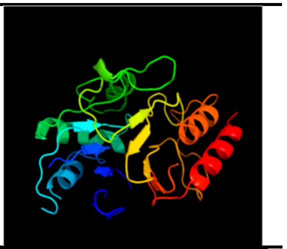
**Class C-like β-lactamase**	**653**	Structure of the octameric penicillin-binding protein (PBP) homologue from *pyrococcus abyssi*	c2qmiH	100%	66%	430 residues (64%) have been modelled with 100% confidence by the single highest scoring template	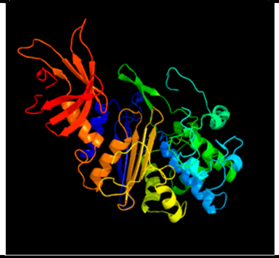

Table 3. Antibiotic Resistance Pattern of *Methanosarcina* Compared to *Elizabethkingia* GOB-15.

	M. Barkeri **Class B**	***Methanosarcina*** **sp. Class C-Like**	***Elizabethkingia*** **GOB-13** [13]
β-lactams			
Ampicillin	R	R	R
Ampicillin/sulbactam	R	R	R
Penicillin	R	R	R
Piperacillin	R	R	R
Piperacillin/tazobactam	R	R	R
Cefoxitin	R	R	R
Ceftriaxone	R	R	R
Ceftazidime	R	R	R
Imipenem	R	R	R
Meropenem	R	R	R
Aztreonam	R	R	R
Non β-lactams			
Gentamicin	R	R	S
Ciprofloxacin	R	R	R
Amikacin	R	R	S
Trimethoprim-sulfamethoxazole	R	R	R

Nd - not determined. R, resistance; S, Susceptible. *Methanosarcina* isolates were culture on SAB medium (Khelaifia *et al.* PLoS One 17;8(4) (2013). 13 - Opota et al., IJAA 49, 93-97 (2016).

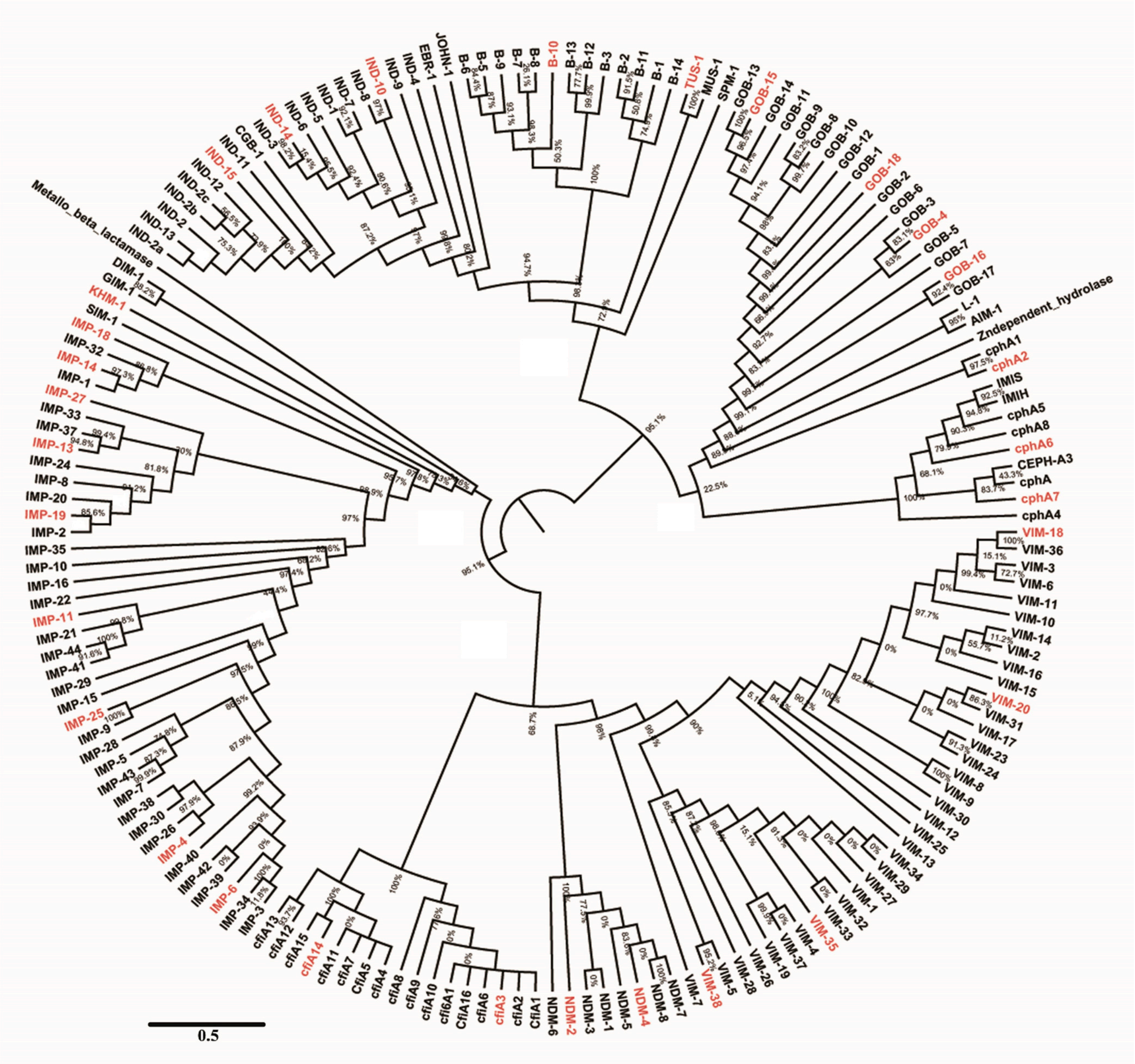
Metallo_beta_lactamase
DIM-1
GIM-1
KHM-1
SIM-1
IMP-18
IMP-32
IMP-14
IMP-1
IMP-27
IMP-33
IMP-37
IMP-13
IMP-24
IMP-8
IMP-20
IMP-19
IMP-2
IMP-35
IMP-10
IMP-16
IMP-22
IMP-11
IMP-21
IMP-44
IMP-41
IMP-29
IMP-15
IMP-25
IMP-9
IMP-28
IMP-5
IMP-43
IMP-7
IMP-38
IMP-30
IMP-26
IMP-4
IMP-40
IMP-42
IMP-39
IMP-6
IMP-34
IMP-3
cfiA13
cfiA12
cfiA15
cfiA14
cfiA11
cfiA7
CfiA5
cfiA4
cfiA8
cfiA9
cfiA10
cfi6A1
CfiA16
cfiA6
cfiA3
cfiA2
CfiA1
NDM-6
NDM-2
NDM-3
NDM-1
NDM-5
NDM-4
NDM-8
NDM-7
VIM-7
VIM-38
VIM-5
VIM-28
VIM-26
VIM-19
VIM-37
VIM-4
VIM-35
VIM-33
VIM-32
VIM-1
VIM-27
VIM-29
VIM-34
VIM-13
VIM-25
VIM-12
VIM-30
VIM-9
VIM-8
VIM-24
VIM-23
VIM-17
VIM-31
VIM-20
VIM-15
VIM-16
VIM-2
VIM-14
VIM-10
VIM-11
VIM-6
VIM-3
VIM-36
VIM-18
cphA4
cphA7
cphA
CEPH-A3
cphA6
cphA8
cphA5
IMIH
IMIS
cphA2
cphA1
Zndependent_hydrolase
AIM-1
L-1
GOB-17
GOB-16
GOB-7
GOB-5
GOB-4
GOB-3
GOB-6
GOB-2
GOB-18
GOB-1
GOB-12
GOB-10
GOB-8
GOB-9
GOB-11
GOB-14
GOB-15
GOB-13
SPM-1
MUS-1
TUS-1
B-14
B-1
B-11
B-2
B-3
B-12
B-13
B-10
B-8
B-7
B-9
B-5
B-6
JOHN-1
EBR-1
IND-4
IND-9
IND-10
IND-8
IND-7
IND-1
IND-5
IND-6
IND-14
IND-3
CGB-1
IND-11
IND-15
IND-12
IND-2c
IND-2b
IND-2
IND-13
IND-2a
0.5

Figure S1. Phylogenetic Tree of Class B β-lactamases: The phylogenetic tree was inferred using the approximate Maximum Likelihood method under the JTT matrix-based model. The analysis involved 174 amino acid sequences from class B β-lactamases. Evolutionary analysis was conducted in FastTree and visualized in FigTree. The existing clades are labeled as b-1 to b-4.

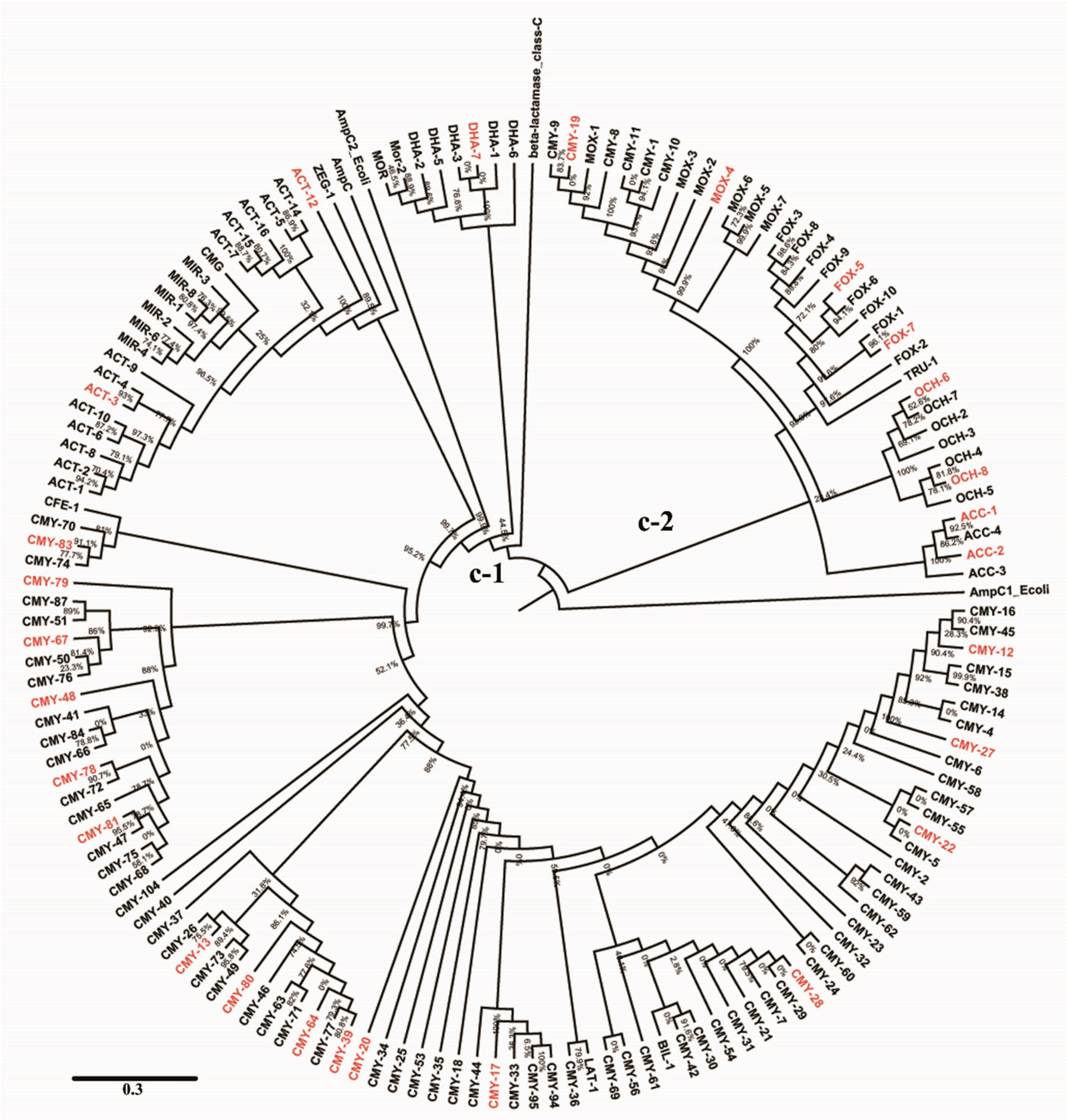

c-1
c-2
0.3
beta-lactamase_class-C
DHA-6
DHA-1
DHA-7
DHA-3
DHA-5
DHA-2
Mor-2
MOR
AmpC2_Ecoli
AmpC
ZEG-1
ACT-12
ACT-14
ACT-5
ACT-16
ACT-15
ACT-7
CMG
MIR-3
MIR-8
MIR-1
MIR-2
MIR-6
MIR-4
ACT-9
ACT-4
ACT-3
ACT-10
ACT-6
ACT-8
ACT-2
ACT-1
CFE-1
CMY-70
CMY-83
CMY-74
CMY-79
CMY-87
CMY-51
CMY-67
CMY-50
CMY-76
CMY-48
CMY-41
CMY-84
CMY-66
CMY-78
CMY-72
CMY-65
CMY-81
CMY-47
CMY-75
CMY-68
CMY-104
CMY-40
CMY-37
CMY-26
CMY-13
CMY-73
CMY-49
CMY-80
CMY-46
CMY-63
CMY-71
CMY-64
CMY-77
CMY-39
CMY-20
CMY-34
CMY-25
CMY-53
CMY-35
CMY-18
CMY-44
CMY-17
CMY-33
CMY-95
CMY-94
CMY-36
LAT-1
CMY-69
CMY-56
CMY-61
BIL-1
CMY-42
CMY-30
CMY-54
CMY-31
CMY-21
CMY-7
CMY-29
CMY-28
CMY-24
CMY-60
CMY-32
CMY-23
CMY-62
CMY-59
CMY-43
CMY-2
CMY-5
CMY-22
CMY-55
CMY-57
CMY-58
CMY-6
CMY-27
CMY-4
CMY-14
CMY-38
CMY-15
CMY-12
CMY-45
CMY-16
AmpC1_Ecoli
ACC-3
ACC-2
ACC-4
ACC-1
OCH-5
OCH-8
OCH-4
OCH-3
OCH-2
OCH-7
OCH-6
TRU-1
FOX-2
FOX-7
FOX-1
FOX-10
FOX-6
FOX-5
FOX-9
FOX-4
FOX-8
FOX-3
MOX-7
MOX-5
MOX-6
MOX-4
MOX-2
MOX-3
CMY-10
CMY-1
CMY-11
CMY-8
MOX-1
CMY-19
CMY-9

Figure S2. Phylogenetic tree of Class C-like β-lactamases: The phylogenetic tree was inferred using the approximate Maximum Likelihood method under the JTT matrix-based model. The analysis involved 151 amino acid sequences from the class C-like β-lactamases. Evolutionary analysis was conducted in FastTree and visualized in FigTree. The existing clades are labeled as c-1 and c-2.

		His118 ↓ Asp120 ↓	His196 ↓	His263 ↓
VIM-18 *Pseudomonas aeruginosa*	S P L A Q	A V H F H D D R V G	G A A H S T L	V I P G H G L P
VIM-20 *Pseudomonas aeruginosa*	S P L A Q	A V H F H D D R V G	G A A H S T L	V I P G H G L P
VIM-35 *Klebsiella oxytoca*	S P L A Q	A V H F H D D R V G	G A A H S T L	V I P G H G L P
VIM-38 *Pseudomonas aeruginosa*	S P L A Q	A V H F H D D R V G	G A A H S T L	V I P G H G L P
NDM-1 *Klebsiella pneumoniae*	Q Q M E Q	A V H A H Q D K M G	G P G H T S I	I V M S H S A P
NDM-2 *Escherichia coli*	Q Q M E Q	A V H A H Q D K M G	G P G H T S I	I V M S H S A P
NDM-4 *Escherichia coli*	Q Q M E Q	A V H A H Q D K M G	G P G H T S I	I V M S H S A P
KHM-1 *Citrobacter freundii*	D S L P K	S I H F H T D S T G	G A G H T P L	V V P G H G K V
IMP-11 *Acinetobacter baumannii*	A S L P K	S I H F H S D S T G	G P G H T Q V	V V P S H S D I
IMP-4 *Pseudomonas aeruginosa*	E P L P K	S I H F H S D S T G	G P G H T P L	V V P S H S E A
IMP-13 *Pseudomonas monteilii*	A A L P K	T I H F H S D S T G	G P G H T Q L	V V S S H S E K
BlaB-10 *Elizabethkingia meningoseptica*	Q Q N P K	N I H S H D D R A G	G K G H T A V	V V A G H D D W
IND-14 *Chryseobacterium indologenes*	A Q V K P	V F H S H D D R A G	G E G H T A V	V I P G H D E W
IND-15 *Chryseobacterium indologenes*	A Q V K P	V F H S H D D R A G	G E G H T V V	V I P G H D E W
gi|851312700|*Methanosarcina barkeri*	M E V R Y	I V H C H Y D H A A	T P G H S K I	L Y P G H G A P
gi|851225341|*Methanosarcina barkeri*	M E V R Y	I V H C H Y D H A A	T P G H S K I	L Y P G H G A P
gi|851262085|*Methanosarcina horonobensis*	M E V R Y	I V H C H Y D H T A	T P G H S K I	L Y S G H G A P
GOB-16 *Elizabethkingia meningoseptica*	E N M P K	L L Q A H Y D H T G	H P G H T K C	W V A S H A S Q
GOB-4 *Elizabethkingia meningoseptica*	E N M P K	L L Q A H Y D H T G	H P G H T K C	W V A S H A S Q
GOB-15 *Elizabethkingia meningoseptica*	E N T N E	L L Q A H Y D H T G	H P G H T K C	W V A S H A S Q
GOB-18 *Elizabethkingia meningoseptica*	E N M P K	L L Q A H Y D H T G	H P G H T K C	W V A S H A S Q

Figure S3. Protein sequences alignment of bacterial and archaeal class B β-lactamases. Known and conserved residues of bacterial metallo-β-lactamases are highlighted with yellow color.

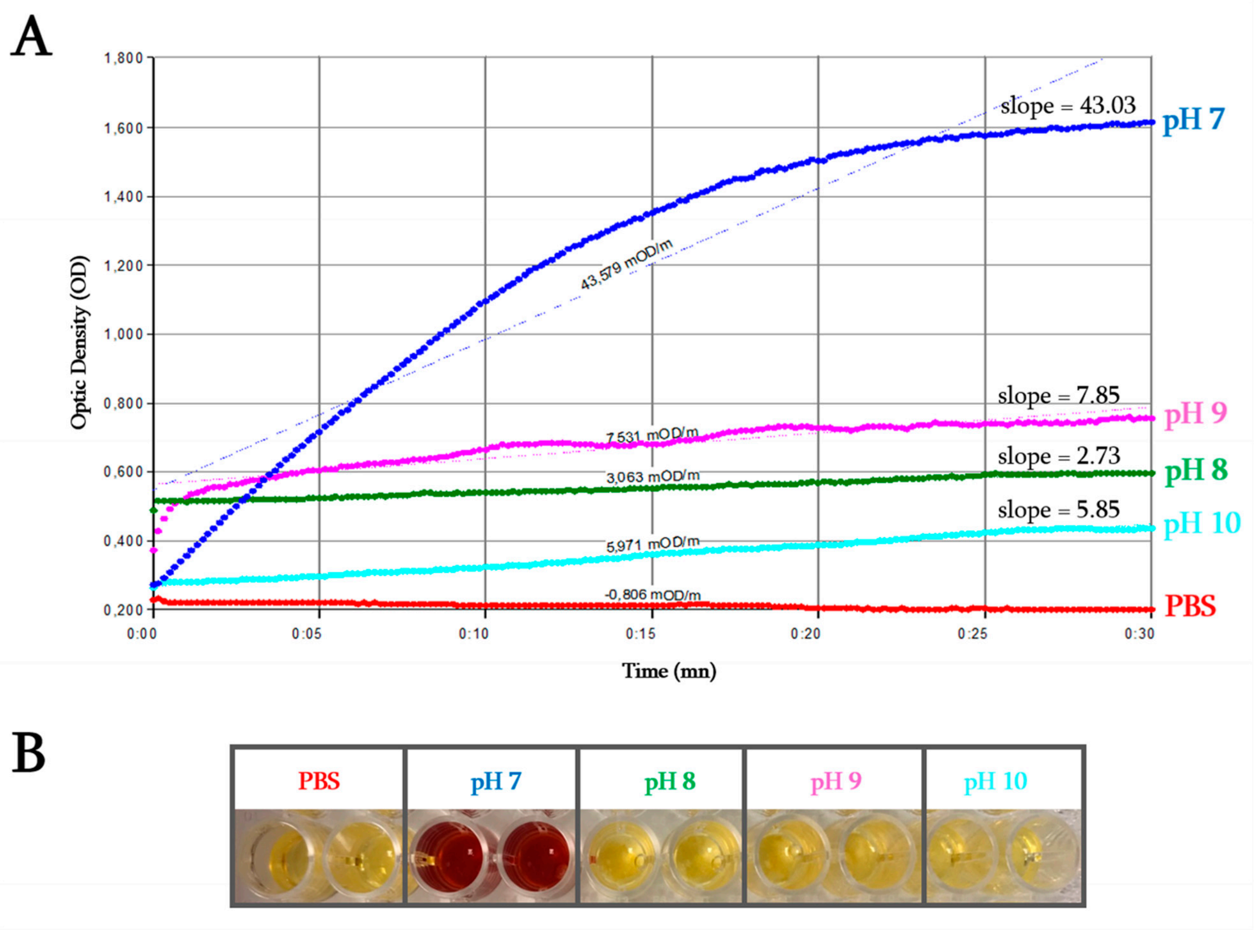

Figure S4. Evaluation of the archaeal MetbaB enzyme activity on nitrocefin at different pH. **(A)** The monitoring of the nitrocefin degradation by MetbaB enzymes during 30 minutes under the four different pH conditions. **(B)** The MetbaB activity test on the chromogenic cephalosporin substrate in liquid medium. .

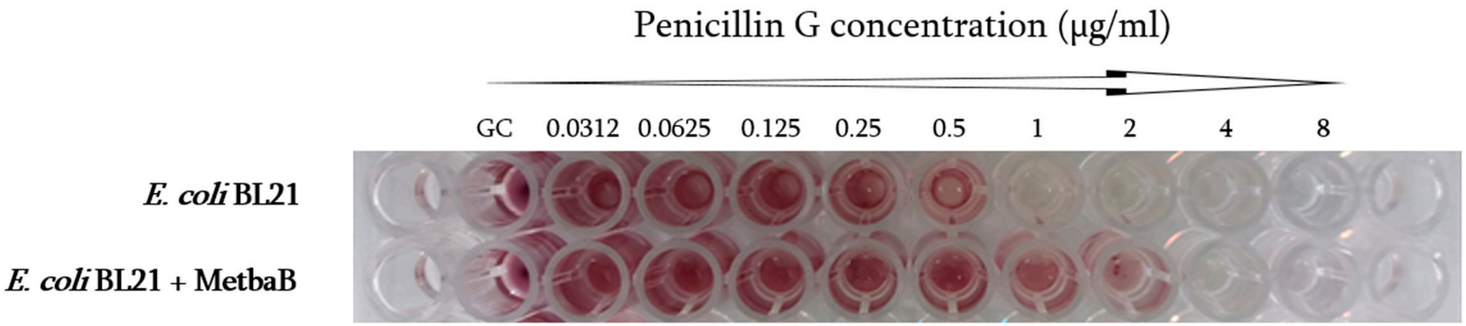

Figure S5. The antibiotic susceptibility testing of a recombinant *E. coli* BL21 mutant containing this Archaeal β-lactamase (*metbaB* gene). GC: Growth control. Bacterial growth is indicated by red color observed in each well.

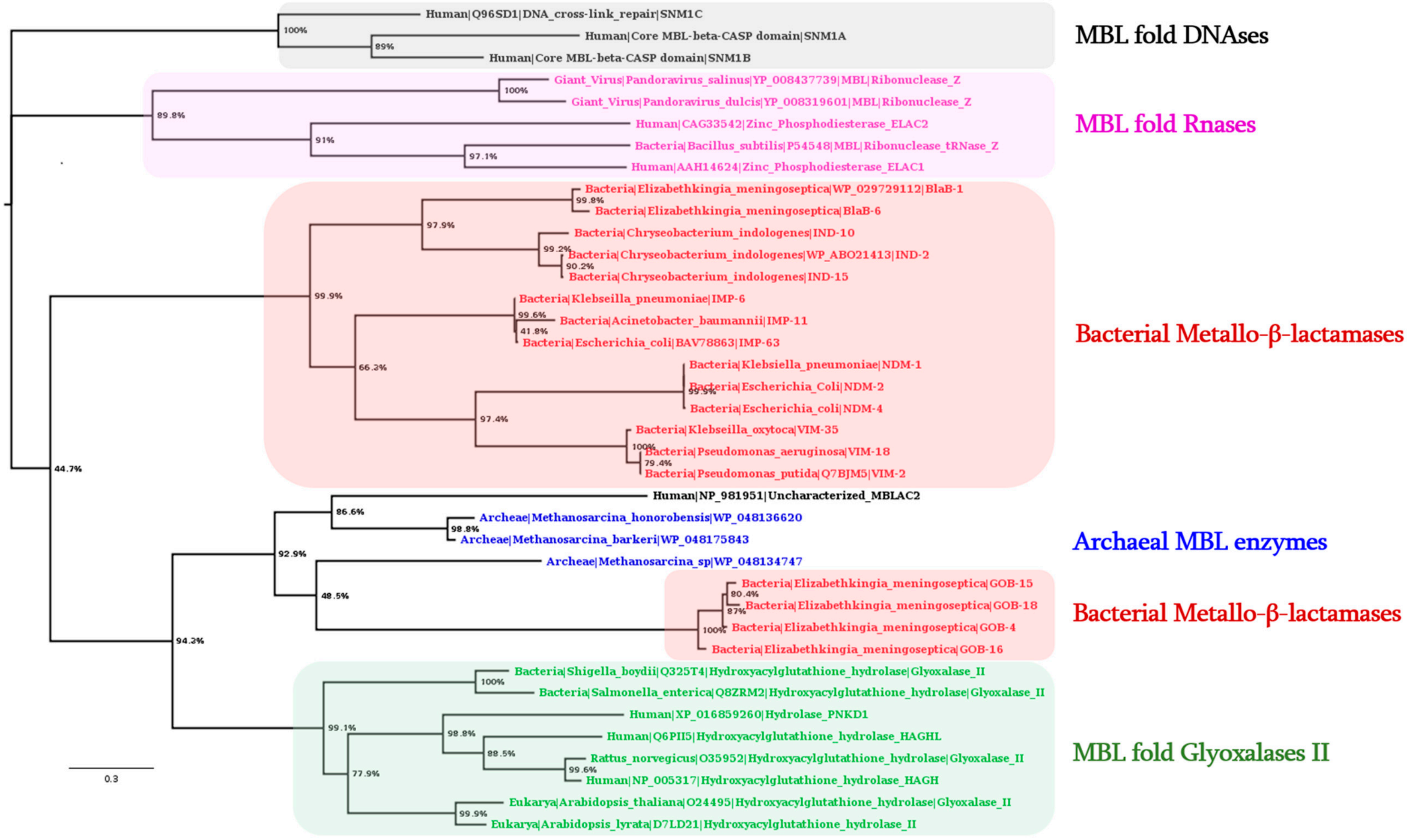

Figure S6. Phylogenetic relation cheap of different MBL fold proteins including β-lactamases, nucleases, ribonucleases, glyoxalase, and identified enzymes from human, bacteria, archaea, and giant virus.

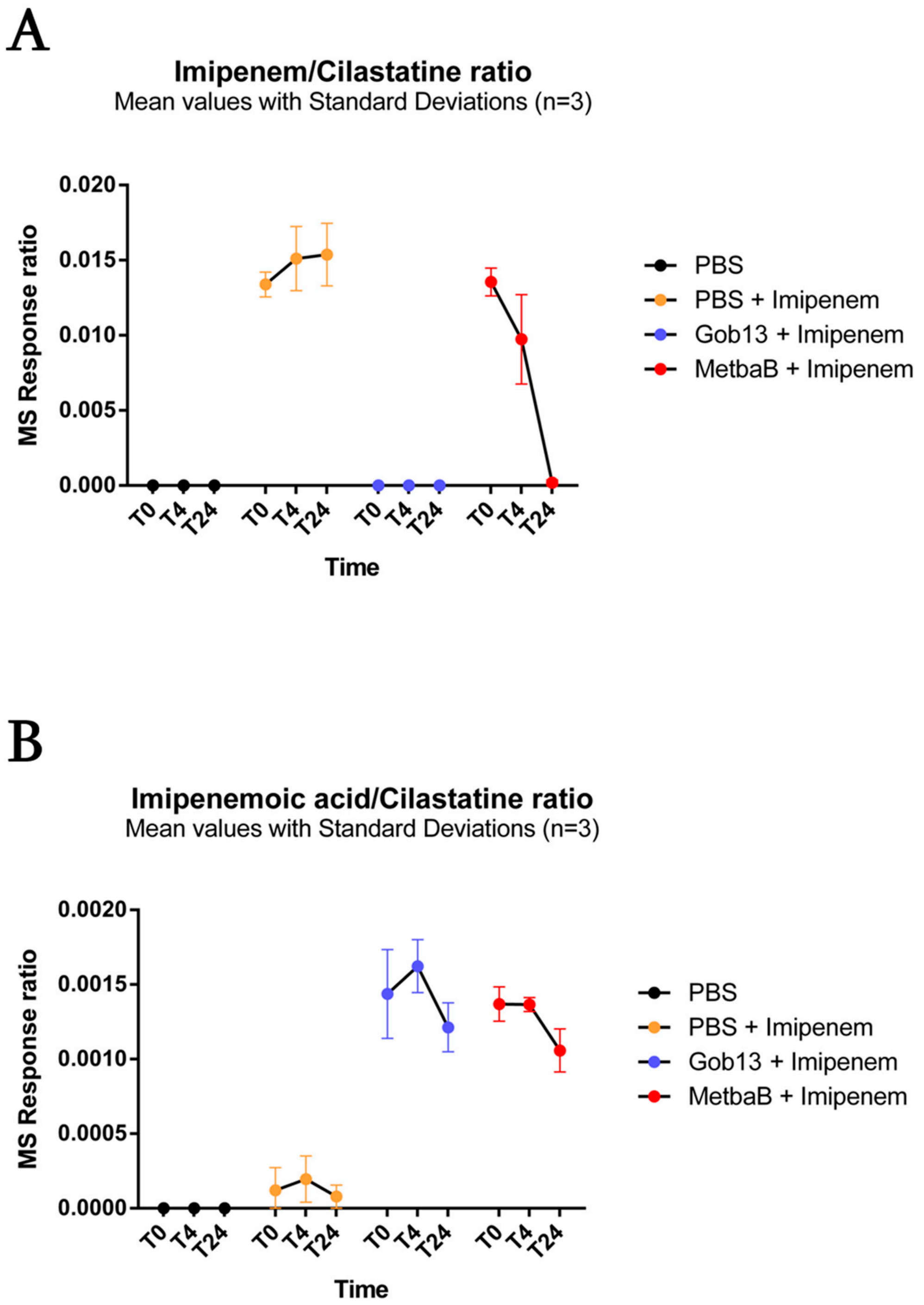

Figure S7. Monitoring the imipenem hydrolysis by the *E. meningoseptica* metallo-β-lactamase GOB-13 and MetbaB enzyme by LC-MS. Time depended production of the metabolite of imipenem (i.e. imipenemoic acid) is measured here using a unhydrolyzed cilastatine substrate. Both enzymes hydrolyze efficiently imipenem through the increase accumulation of its metabolite.

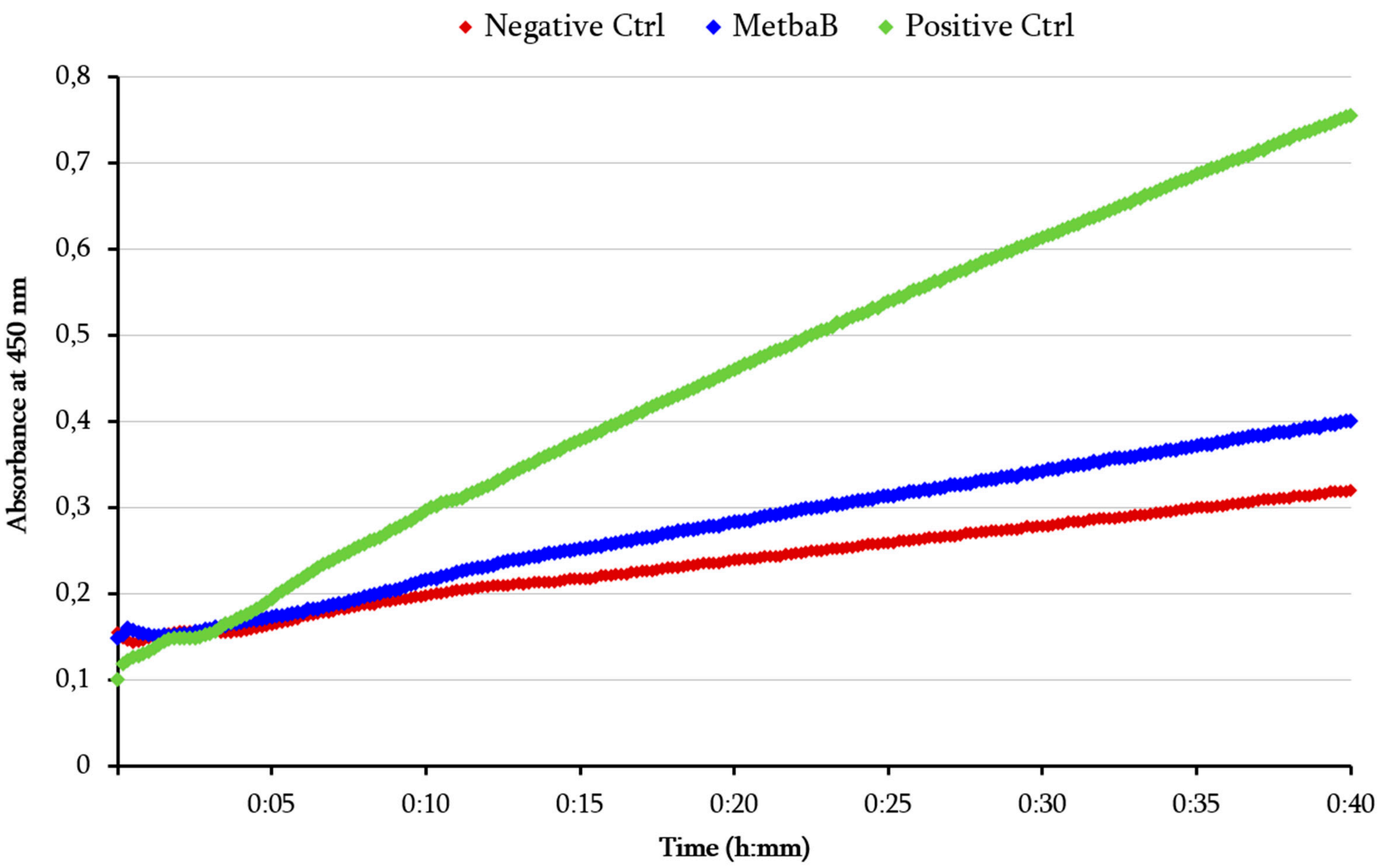

Figure S8. Glyoxalase II activity assay. D-lactate production was monitored for 40 min following absorbance variations at 450 nm. The GloII positive control was provided in the "Glyoxalase II Activity kit" from BioVision. The negative control was the reagent background control (same mixture with no active enzyme).

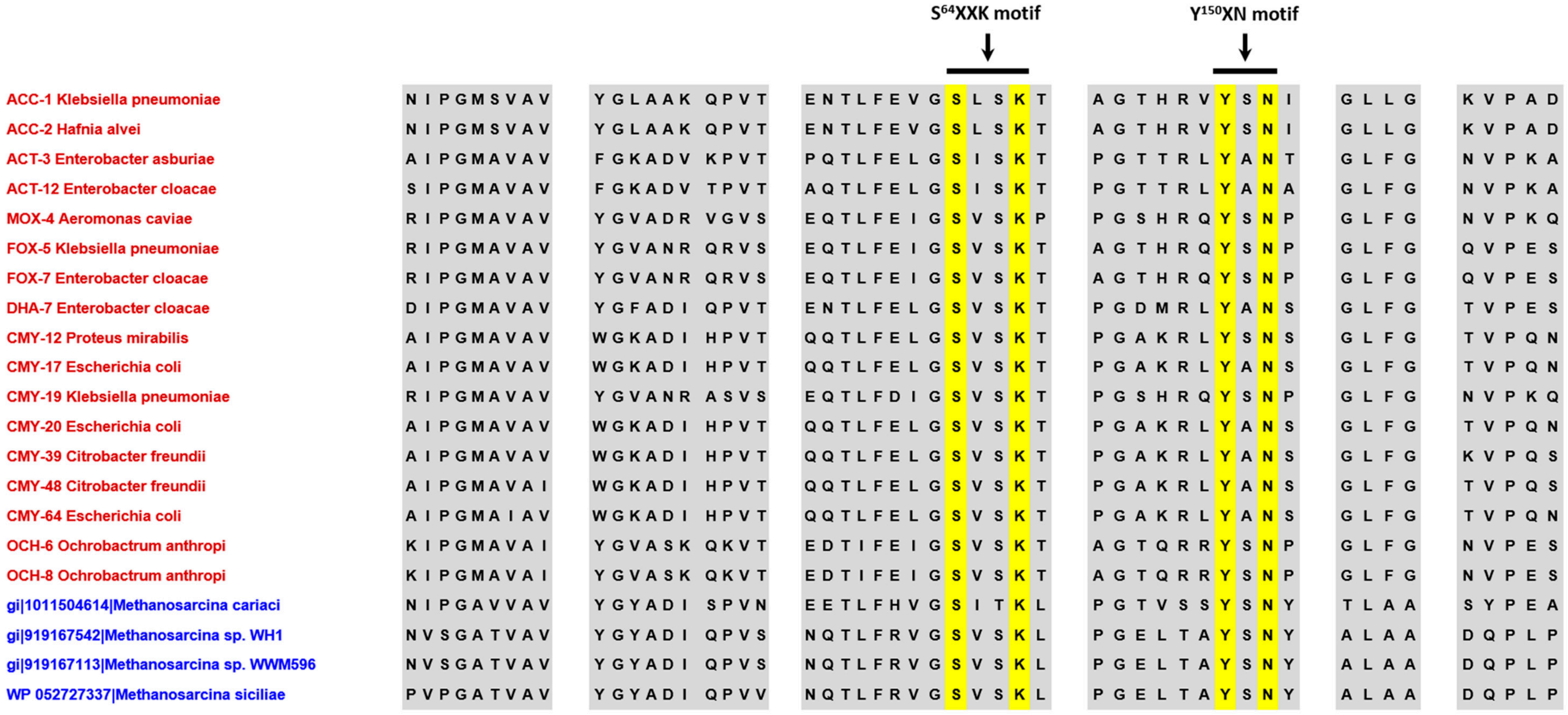

Figure S9. Protein alignment sequences of bacterial and archaeal class C-like β-lactamase proteins. Known and conserved motifs of bacterial class C β-lactamases are highlighted with yellow color.

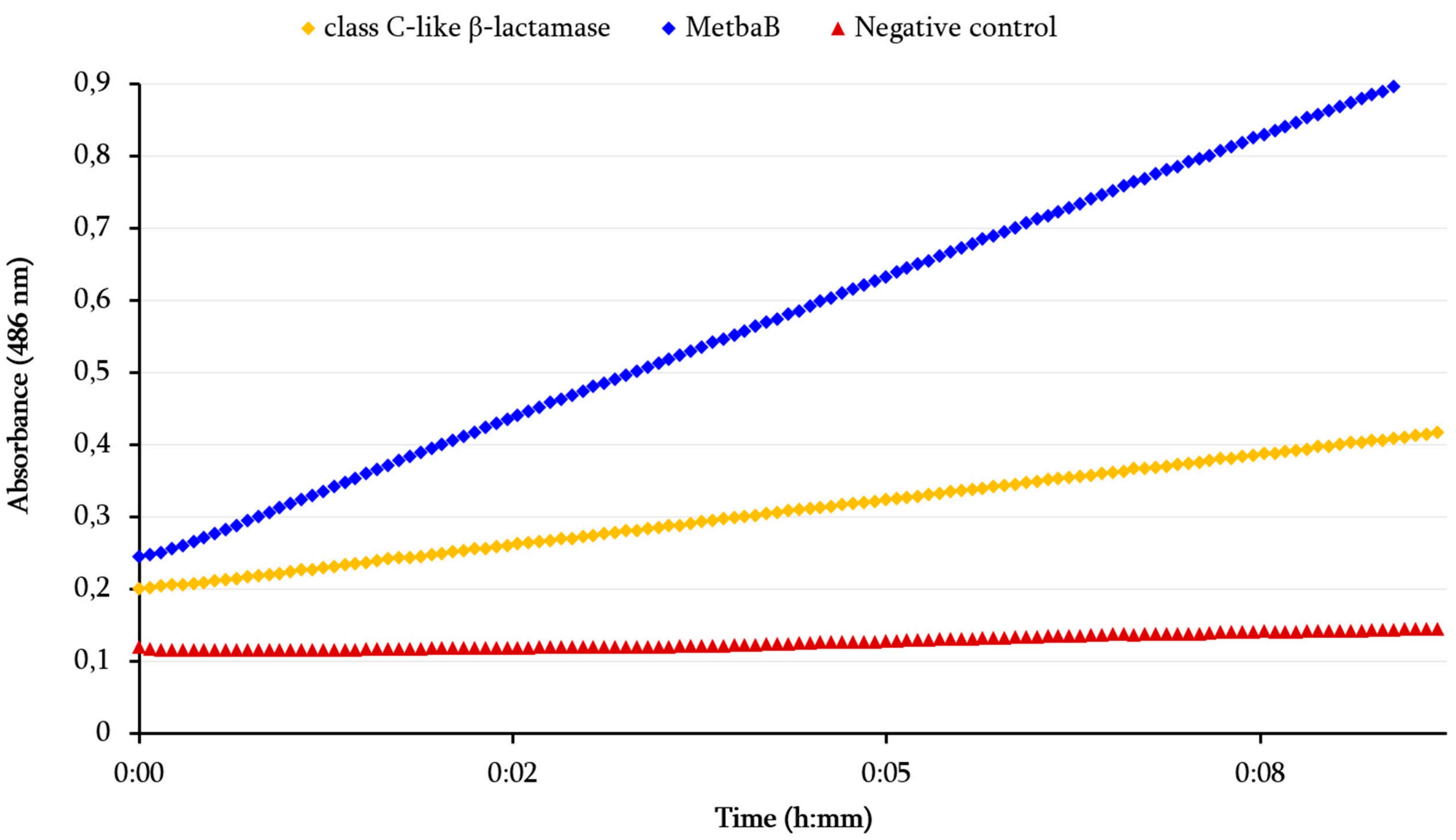

Figure S10. Degradation of nitrocefin by MetbaB and the archaeal class C-like β-lactamases was monitored for 10 min following absorbance variations at 486 nm. Enzymes were respectively kept at concentrations of 10 μM and 4 μM for this assay. The negative control displayed here was the same reagent mixture without any active enzyme.

life

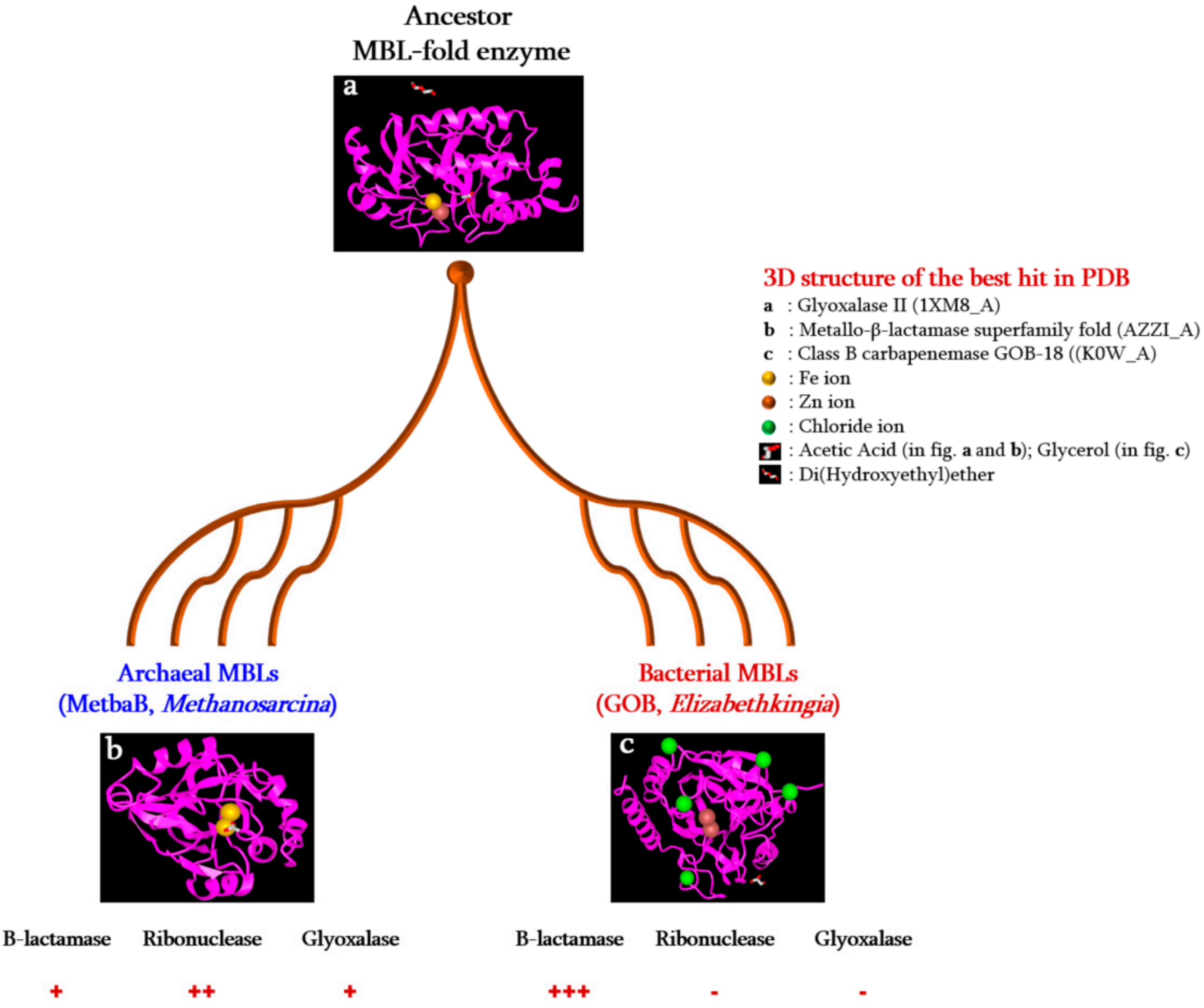

Figure S11. Putative evolution scenario of enzymatic activities of MBL fold proteins. (+): positive activity; (-) : negative activity.

Publisher's Note: MDPI stays neutral with regard to jurisdictional claims in published maps and institutional affiliations.

© 2020 by the authors. Submitted for possible open access publication under the terms and conditions of the Creative Commons Attribution (CC BY) license (http://creativecommons.org/licenses/by/4.0/).
